# Supplementary material for: Content-rich biological network constructed by mining PubMed abstracts
Source: BMC Bioinformatics. 2004 Oct 8;5:147. doi: 10.1186/1471-2105-5-147 (PMC528731; doi:10.1186/1471-2105-5-147)
Supplement: Additional File 2 — The original results of the above study (non-essential files are deleted to keep the file size under the limit set by BMC bioinformatics). [file 1471-2105-5-147-S2.bz2 › chilibotAdditionalFile2/dip10/60ID7538672E215/html/PRDM2_RB1.html]

 


 **PRDM2** and **RB1** 
  
Found 23 abstracts in PubMed, retrieved 10.  
 

 What does Google say? 
 PDF only 
| .edu only 

---

**Interactive relationship** (e.g. stimulation, inhibition, etc)

**Inhibitory relationship**- BACKGROUND The retinoblastoma  [ **RB1** ]  interacting zinc finger gene RIZ  [ **PRDM2** ]  is expressed in two forms RIZ1  [ **PRDM2** ]  and RIZ2  [ **PRDM2** ]  that differ for the presence near the N terminus of RIZ1  [ **PRDM2** ]  of a conserved domain, defined PR PRDI BF1 RIZ  [ **PRDM2** ]  homology, homologous to a similar domain present in other proteins recognized as tumor suppressor gene products.  Ref: 11591891 Mol Med, 2001
- Recently, the retinoblastoma  [ **RB1** ]  protein interacting zinc finger gene RIZ  [ **PRDM2** ]  has been proposed as a candidate for the tumor suppressor locus on 1p36, because of the common loss of RIZ1  [ **PRDM2** ]  RNA in human tumors.  Ref: 12082534 Oncogene, 2002
**Neutral relationship**

**Non-interactive relationship** (e.g. studied together, co-existance, homology, etc.)

- Many lines of evidence suggest that the retinoblastoma  [ **RB1** ]  protein interacting zinc finger gene RIZ  [ **PRDM2** ]  is a strong candidate for the tumor suppressor locus on 1p36, a region commonly deleted in many human cancers with chromosomal instability.  Ref: 10987271 Cancer Res, 2000
- The retinoblastoma  [ **RB1** ]  protein interacting zinc finger gene RIZ  [ **PRDM2** ] , a member of the nuclear protein methyltransferase superfamily, is characterized by the presence of the N terminal PR domain.  Ref: 12472571 Br J Haematol, 2002
- Altered expression of retinoblastoma  [ **RB1** ]  protein interacting zinc finger gene, RIZ  [ **PRDM2** ] , in human leukaemia.  Ref: 12472571 Br J Haematol, 2002
- The retinoblastoma  [ **RB1** ]  protein interacting zinc finger gene RIZ1  [ **PRDM2** ]  is a tumor suppressor gene and a member of a nuclear histone protein methyltransferase superfamily.  Ref: 11719434 Cancer Res, 2001
- The retinoblastoma  [ **RB1** ]  protein interacting zinc finger gene RIZ  [ **PRDM2** ]   **PRDM2**  is a member, by sequence homology, of a nuclear protein methyltransferase MTase superfamily involved in chromatin mediated gene expression.  Ref: 11544182 Genes Dev, 2001
- RIZ  [ **PRDM2** ] , the retinoblastoma  [ **RB1** ]  protein interacting zinc finger gene, is mutated in genetically unstable cancers of the pancreas, stomach, and colorectum.  Ref: 11135439 Genes Chromosomes Cancer, 2001
- Similar bipartite sequences can be found in other viral and cellular proteins that can interact with CtBP, including the retinoblastoma  [ **RB1** ]  interacting protein methyltransferase RIZ  [ **PRDM2** ] , the oncoprotein EVI1, and Marek s disease virus transforming protein Meq.  Ref: 12372828 J Biol Chem, 2002
- The retinoblastoma  [ **RB1** ]  protein interacting zinc finger RIZ  [ **PRDM2** ]  gene is a candidate tumor suppressor gene on 1p36, a region frequently rearranged in a wide variety of human tumors.  Ref: 11135439 Genes Chromosomes Cancer, 2001
